# Factors associated with young adults' knowledge regarding family history of Stroke^1^

**DOI:** 10.1590/1518-8345.1285.2814

**Published:** 2016-11-21

**Authors:** Maria Jose Melo Ramos Lima, Thereza Maria Magalhães Moreira, Raquel Sampaio Florêncio, Predro Braga

**Affiliations:** 2 Master's student, Universidade Estadual do Ceará, Fortaleza, CE, Brazil.; 3 PhD, Adjunct Professor, Universidade Estadual do Ceará, Fortaleza, CE, Brazil.; 4 Doctoral student, Universidade Estadual do Ceará, Fortaleza, CE, Brazil. Scholarship holder from Coordenação de Aperfeiçoamento de Pessoal em Nível Superior (CAPES), Brazil.

**Keywords:** Cardiovascular Diseases, Stroke, Young Adult, Heredity, Health Knowledge, Attitudes, Practice, Prevention

## Abstract

**Objective:**

to analyze the factors associated with young adults' knowledge regarding family history of stroke.

**Method:**

an analytical transversal study, with 579 young adults from state schools, with collection of sociodemographic, clinical and risk factor-related variables, analyzed using logistic regression (backward elimination).

**Results:**

a statistical association was detected between age, civil status, and classification of arterial blood pressure and abdominal circumference with knowledge of family history of stroke. In the final logistic regression model, a statistical association was observed between knowledge regarding family history of stroke and the civil status of having a partner (ORa=1.61[1.07-2.42]; p=0.023), abdominal circumference (ORa=0.98[0.96-0.99]; p=0.012) and normal arterial blood pressure (ORa=2.56[1.19-5.52]; p=0.016).

**Conclusion:**

an association was observed between socioeconomic factors and risk factors for stroke and knowledge of family history of stroke, suggesting the need for health education or even educational programs on this topic for the clientele in question.

## Introduction

Stroke is an important public health problem worldwide, as it causes physical, functional and emotional sequelae. Furthermore, it is the principal cause of death and disability, with major economic and social impact^1^ ^-^ ^2^.

Between the end of the 1970s and 2008, the age-adjusted rate of incidence of stroke worldwide reduced by 42% in high-income countries. Nevertheless, it remains the fourth largest cause of death in the United States. Currently it is the main cause of death in the majority of Latin American countries, and Brazil has a high prevalence. The majority of risk factors are clearly avoidable, which would prevent various deaths and disabilities^3^ ^-^ ^4^.

Studies on the epidemiology of the cardiovascular diseases (CVD) in Brazil have evidenced that these have been studied little, as has the existence of disparities between the regions; in their turn, they show that mortality by stroke is higher in the poorer regions of Brazil, such as the North-East, which is to a large extent attributable to social factors such as low standards of care^3^ ^,^ ^5^ ^-^ ^6^.

In the developing countries, such as Brazil, young adults are particularly vulnerable to the chronic diseases. They are commonly exposed to risks, such as socioeconomic difficulties and environmental and social factors, as well as biological and hereditary factors - and tend to develop precocious CVD. The World Health Organization (WHO) and the Pan-American Health Organization (PAHO) estimate that exposure to cardiovascular risk factors led to 36 million premature deaths between 2011 and 2015^7^.

Various studies have presented evidence that CVD manifested in the adult age results from a complex interaction between risk factors which may originate in youth^8^. Many of these factors can be prevented, as they are modifiable. However, there are nonmodifiable factors, such as age, gender, race and family history (FH) of CVD^9^. 

The history of the family's health is a useful tool for understanding risks to health and preventing illnesses in individuals and their close relatives^10^. The FH is a nonmodifiable risk factor for the development of stroke, through its link with genetic factors. The literature indicates that the FH and hypertension are strongly associated with higher incidence of stroke, particularly in families of African or mixed African descent^11^.

The study is relevant, as knowledge regarding FH of illnesses offers the opportunity to undertake health promotion and intervention studies in high-risk groups, particularly in countries of mid- or low income, as mortality from stroke is higher in these countries. 

In this regard, considering this health issue's repercussions in the lives of those who suffer it, the adoption is proposed of the stroke prevention quadrilateral. This covers a joint network of four pillars: demographic surveillance and a research network of the course, community-based primary and secondary prevention programs, and the implantation of acute stroke care units and neuro-rehabilitation centers^12^. 

Starting from the presupposition that family antecedents for CVD can influence the adoption of a healthy lifestyle by young adults, it is understood that the greater the knowledge that the young person has of his or her antecedents, the more sensible the self-care practices and prevention of risk factors for stroke will be. As a result, identifying the factors involved in this knowledge may support interventions undertaken by nurses and other members of the multi-professional team in order to favor and improve these young people's lifestyle. Therefore, this study's objective was to analyze the factors associated with young adults' knowledge regarding family history of stroke. 

## Method

This is an analytical transversal quantitative study, undertaken in the city of Fortaleza in the Brazilian state of Ceará (CE), in the schools of the State Department for Education of Ceará (SEDUC). This study is part of the umbrella project titled "Analysis of overweight/obesity and its association with cardiovascular health in young adults attending school in a capital of the Brazilian Northeast: support for health education undertaken by the nurse". 

For this study, the sample was made up from the population of young adults attending school in the municipality, aged between 20 and 24 years old, who were enrolled in normal state schools or schools for young people and adults.

Considering that the number of young people in school was unknown, the sample was defined based on the calculation for infinite populations. For the purposes of the calculation, the decision was made to take into account the data for determination of the prevalence of the phenomenon: young adults' knowledge regarding FH of stroke. After this analysis, the prevalence of the phenomenon obtained was 18.0%, a value incorporated for calculation of the sample, defined by the following formula: n = (*z* ^2^_5%_ xP x Q)/e^2^, where: *n* is the sample; *z* is the distribution value, at a level of significance of 5% (1.96); *P* is the prevalence of the phenomenon (18.0%); *Q*(82.0%) is the complementary percentage of *P* (Q = 100 - P); and *e* is the sampling error (3.5%).

Based on the result of the calculation, the authors arrived at a sample of 463 young adults attending school. However, in order to avoid possible losses, an addition of 25% was made on top of the total value, thus obtaining a final sample of 579 students. These were from 26 schools located in six regional units of the municipality. The schools were selected randomly and the students by convenience. 

The following were considered as inclusion criteria for the sample: to be officially enrolled in the schools studied, to be in the age range between 20 and 24 years old, and to be present on the days of data collection. The exclusion criteria was pregnancy, as this would make it impossible to measure abdominal circumference. 

Data collection was undertaken between October 2013 and October 2014, immediately after the training of a multi-professional team. This was made up of 10 health professionals (nurses, physiotherapists and physical education professionals) and five students of nursing. After the training, the collection covered three phases: 1) raising the awareness, and selection, of the participants by school; 2) administration of a questionnaire for collection of data referent to the following variables: young adults' knowledge regarding family history of stroke, socioeconomic situation, self perception of health, lifestyle and risk factors for stroke; and 3) undertaking examinations to obtain data regarding clinical characteristics observed (Presence of high blood pressure, biochemical tests: glycemia and total cholesterol); Body Mass Index (BMI) and abdominal circumference (AC). 

The outcome variable considered was "the young adults' knowledge regarding family history of stroke", this categorized as "knows/does not know". At this point, the possible predictive variables considered were: 1) socioeconomic situation: age (in years); sex: male/female; self-reported race: White/others; civil status: with a partner/without a partner; children: no/yes; family income: up to one minimum salary/more than one minimum salary); 2) self perception of health: positive (good or excellent)/negative (normal or bad); 3) lifestyle and risk factors for stroke (currently smokes: no/yes; consumes alcohol: no/yes; practices physical activity: no (less than 150 minutes of activity per week)/yes (150 minutes or more of activity per week); balanced diet: no/yes; consumes large amounts of salt and sugar: no/yes; stress: no/yes; uses illicit drugs: sometimes/never; classification of blood pressure: normal (good or normal)/altered (borderline, stage one, two or three SAH, or isolated systolic hypertension); nutritional status: normal (BMI > 18.5 to < 25 kg/m^2)^/overweight or obesity (BMI > 25 kg/m^2)^; abdominal circumference (in centimeters); casual glycemia (in milligrams/deciliters); total cholesterol (in milligrams/deciliters). 

Arterial blood pressure was checked by the indirect method using the auscultatory technique and a calibrated aneroid sphygmomanometer. The technique for checking and assessment of the numbers for blood pressure followed the protocol recommended by the Brazilian Society of Cardiology in the Brazilian Hypertension Guidelines^13^.

The anthropometric measurements were undertaken in a standardized way. For height, the examinees stood upright, barefoot, with their feet together and the arms by their sides, and a non-stretch tape measure was used, attached to a wall which had no skirting board. The measurement of Abdominal Circumference or of the waist was undertaken using a non-stretch tape measure and with the clothes out of the way, the tape measure being placed at the midpoint between the anterior superior iliac crest and the lowest rib, based in the normal values of 88 cm and 102 cm for women and men, respectively^13^. Weight was determined using a pair of electric scales for adults, with the participant standing, with the arms against the body, barefoot, and bearing as little weight in clothes as possible.

The biochemical indicators for postprandrial glucose and total cholesterol were undertaken using test strips after collecting capillary blood, taking into account aseptic technique and the use of Personal Protective Equipment (PPE), with sharps being discarded in the appropriate way.

After obtaining the data, the association of the categorical variables was undertaken (sex, self-reported race, civil status, children, family income, self perception of health, currently smoking, consumes alcohol, classification of the AP, nutritional status, practice of physical exercise, balanced diet, stress, use of illicit drugs and high consumption of salt and sugar) through nonparametric tests of *^_x2_^* for categorical variables and the Student t-test for numerical variables (age, AC, glucose levels, total cholesterol), adopting a level of statistical significance of 5% (p<0.05), regarding the association of the variables in question. In order to estimate the strength of association, the odds ratio (OR) was calculated, with a confidence interval of 95%. In the analysis, the Statistical Package for the Social Sciences software (SPSS), version 20.0, was used. 

In order to minimize the effects of confounding variables, a logistic regression analysis was undertaken, considering the descriptive level of p<0.20 for inclusion in the initial model. The criteria established in this stage of analysis for the variables to remain in the model was for the Wald test to present at least one category with statistical significance of the p-value < 0.05. The method used for regression was backward elimination. After the analysis, the data were presented as text and in tables, having been discussed in accordance with the relevant literature. 

This study complied with ethical and legal precepts for research involving human beings, and was approved by the Research Ethics Committee of the Ceará State University, in process N. 263.271/2013.

## Results

According to the contents of Tables 1 and 2, socioeconomic similarities were ascertained related to the risk factors between the groups with knowledge and without knowledge regarding family history of stroke. 

**Table 1:**
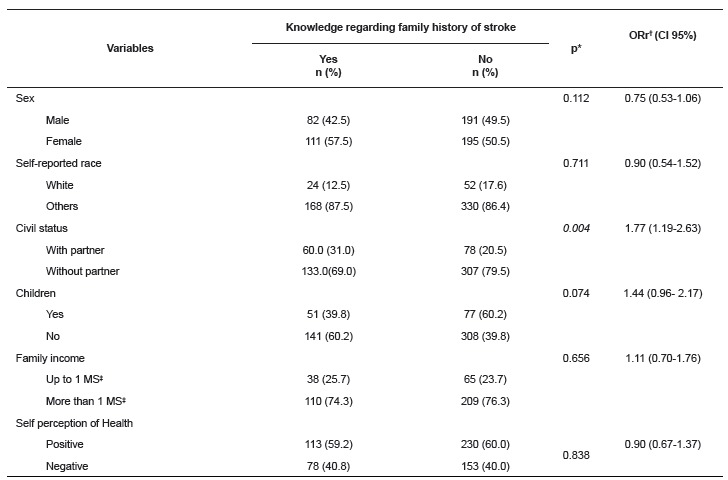
Analysis of the association between sociodemographic characteristics and young adult students' knowledge regarding family history of stroke. Fortaleza, CE, Brazil, 2014

*Statistical significance of the Pearson Chi Squared test; †ORr: raw odds ratio; ‡MS: minimum salary (R$ 724.00 in 2014, in Brazil)

**Table 2:**
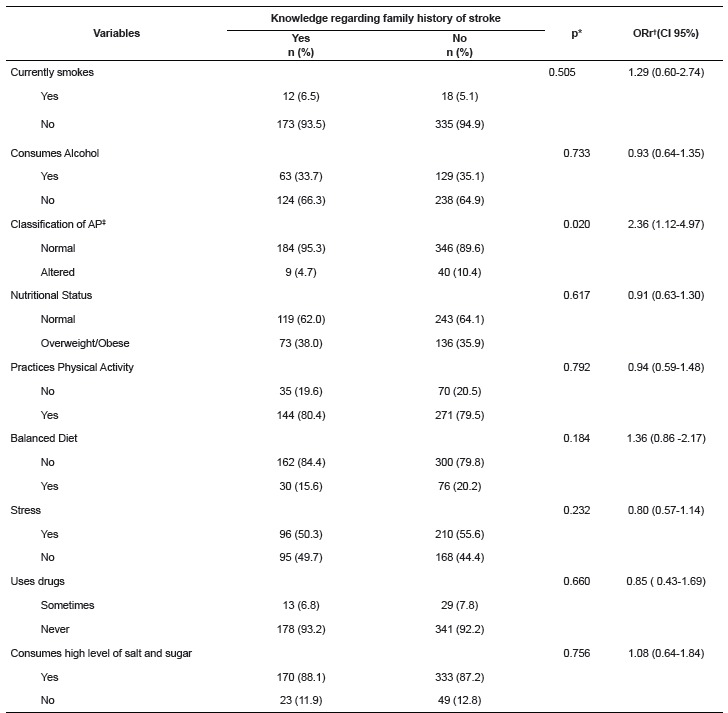
Analysis of the association between risk factors for stroke and knowledge regarding family history of stroke, of young adult students. Fortaleza, CE, Brazil, 2014

*Statistical significance of the Pearson Chi squared test;†ORr: raw odds ratio; ‡AP: arterial blood pressure

It was found that nearly all of the group with knowledge were aged between 20 and 22 years old (98.4%), with a mean age of 22.3 (±1.40); slightly over half were female (57.5%), a large majority stated that they were nonwhite (black, mulatto or mixed race) (87.5%), a large majority lived without a partner (69.0%), and did not have children (60.2%). The predominant family income was over one minimum salary (74.3%) and more than half stated that they perceived their health as positive (59.2%). As shown, these characteristics were similar between the groups studied, except for the variable of "children", for which the large majority of the young people without knowledge of FH of stroke stated that they had children (60.2%).

Still in relation to the socioeconomic characteristics, only the marital situation (p<0.05) presented a statistically significant association with knowledge on FH of stroke, in which the young people with a partner had a 1.77 (1.19-2.63) higher chance of presenting the outcome in question. 

According to Table 2, concerning the group without knowledge, it was ascertained that the large majority did not smoke (93.5%), slightly over half do not drink alcohol (66.3%), and only 4.7% showed high levels of blood pressure (p<0.05). In relation to body weight, slightly over half had normal body weight (62.0%); the large majority practiced physical exercise (80.0%) and did not have a balanced diet (84.4%). In addition to this, nearly all (93.3%) did not use drugs and consumed large quantities of foods with high levels of salt and sugar (88.1%). 

There were similarities between the risk factors in the group with knowledge and without knowledge regarding the FH for stroke, principally when there was not a healthy diet (79.8%) and when there was high consumption of foods rich in salt and sugar (87.2%). 

In the analysis of risk factors for stroke, there was statistically significant association between the outcome of the study and 'classification of arterial blood pressure' (p=0.020). In a complementary way, it was observed that those young adults with arterial blood pressure classified as normal had a 2.36 (1.12-4.97) higher chance of presenting knowledge regarding family history of stroke. 

Below, in Table 3, one can observe that the means for age, AC and total cholesterol were higher in the group of young adults who knew their FH for stroke, in contrast with what was found for glycemia. Based in what was present, a significant statistical association was ascertained of the outcome with 'age' and 'Abdominal circumference'. 

**Table 3:** Analysis of the association between the variables of age, abdominal circumference, glycemia and cholesterol, with young adult students' knowledge regarding family history of stroke. Fortaleza, CE, Brazil, 2014

Variables	Knowledge of family history	Ignorance of family history	Student t-test	p*
	Mean (Standard deviation)	Mean (Standard deviation)		
Age	21.3 (1.45)	21.0 (1.33)	2.341	0.020
AC^†^	83.0 (11.30)	80.7 (10.38)	2.391	0.017
Glycemia	111 (15.5)	112.0 (18.0)	0.650	0.516
Cholesterol	162.0(21.06)	159.8 (18.5)	1.271	0.224

*Statistical significance of the Student t-test; †AC: abdominal circumference.

As shown in Table 4, to enter the initial regression model, the following variables were selected: gender (p=0.112), marital situation (p=0.004), children (p=0.074), age (p=0.020), balanced diet (p=0.184), classification of the AP (p=0.020) and AC (p=0.017).

**Table 4:** Simple Logistic Regression of the variables related to knowledge of family history of stroke. Fortaleza, CE, Brazil, 2014

Variables	Knowledge of family history of Stroke				
	Stage 1	Stage 2	Stage 3	Stage 4	Stage 5
	ORa*(CI 95%)	ORa*(CI 95%)	ORa*(CI 95%)	ORa*(CI 95%)	ORa*(CI 95%)
Age	0.90(0.79-1.03)	0.90(0.79-1.03)	0.90(0.79-1.03)	0.90(0.79-1.02)	-
	p=0.119	p=0.119	p= 0.111	p= 0.109	
Gender	0.94(0.64-.37)	0.94(0.65-1.37)	-	-	-
	p= 0.738	p= 0.751			
Civil status	1.52(0.98-2.36)	1.50(0.99-2.29)	1.52(0.99-2.30)	1.51(0.99-2.29)	1.61(1.07-2.42)
	p=0.060	p=0.057	p= 0.051	p=0.053	p=0.023
Children	0.97(0.61-1.53)	-	-	-	-
	p=0.884				
Balanced Diet	0.92(0.64-1.32)	0.92(0.64-1.32)	0.92(0.64-1.32)	-	-
	p=0.658	p=0.655	p=0.652		
AC^†^	0.98(0.96-0.99)	0.99(0.99-1.01)	0.98(0.96-0.99)	0.98(0.96-0.99)	0.98(0.96-0.99)
	p=0.019	p=0.499	p=0.018	p=0.018	p=0.012
Classification AP^‡^	2.49(1.13-5.47)	2.50(1.13-5.47)	2.56(1.19-5.52)	2.56(1.19-5.53)	2.56(1.19-5.52)
	p=0.023	p= 0.023	p=0.017	p=0.016	p=0.016

*ORa: adjusted odds ratio; †AC: abdominal circumference ; ‡AP: arterial blood pressure

In accordance with the logistic regression model, a statistical association of knowledge regarding family history of stroke with civil status (ORa=1.61[1.07-2.42]; *p*=0.023), abdominal circumference (ORa=0.98[0.96-0.99]; *p*=0.012) and classification of arterial blood pressure (ORa=2.56[1.19-5.52]; *p*=0.016) was ascertained.

## Discussion

The lifestyle adopted by individuals can bring benefits or risks for their health. The act of choosing to adopt healthy practices seems to be related to various factors: the individual's perception in relation to his or her position in life, or cultural context and system of values in which the person lives - his or her objectives, expectations, standards and concerns^14^. Hence, the increasingly early exposure to the various risk factors related to lifestyle influences the cases of stroke in young adults. 

Knowing the FH for health is a useful tool for understanding of risks to health and prevention of illness in individuals and their close relatives. Evidence accumulated over decades demonstrates convincingly that the family history of one of the parents or of a sibling is associated with CVD, which is manifested mainly in the form of stroke^15^.

It is known that the health of a group of individuals is the result of the interaction between genetic factors and various environmental factors, to which lifestyle habits are directly related. Willingness to change can result from various factors: gender, civil status, income, educational level and the various cultural and behavioral habits. 

The health-illness situation has determinants and conditioning factors from highly general factors of a social, economic, or political nature, and the means through which these impact on the health situation of groups and people, there being no simple direct cause-and-effect relationship, influencing all the dimensions of populations' health processes, either from the point of view of the individual or of the community in which he or she is integrated. 

It is understood that the greater the young person's knowledge of his or her antecedents, the more sensible will be the practices of self-care and prevention of risk factors for stroke. As a result, understanding which factors are involved in this knowledge could support interventions for increasing still further the level of information and, as a consequence, improving lifestyle. 

The international and Brazilian scientific literature on knowledge of FH of stroke is scarce. One Brazilian study which evaluated one population's knowledge in a general way regarding stroke ascertained that this was considered inadequate for the large majority^16^. Thus, taking into account that probably the young adult has little knowledge regarding the topic, it becomes difficult to perceive the cases of the illness in the family. As a result, other studies need to be undertaken with this population, in order to identify principally what influences this knowledge. 

As seen in the present study, there are factors associated with knowledge of the FH for stroke. Among these, one finds civil status, which presented a statistically significant association with knowledge regarding the family history of stroke. Despite the Brazilian tendency to show that young adults' seeking to join the job market is related to living in their parents' house, and to entering a union with a partner only after attaining greater financial stability^17^, young adults with a partner presented a higher probability of knowing their FH. 

Individuals' health has a close link to the beliefs, values, relationships, rights and duties of their family system. Hence, it is supposed that the fact that a person is married imposes greater responsibility regarding care, which implies knowing more about the family health situation. 

In relation to the analysis of the students' clinical characteristics, the large majority of the participants presented blood pressure levels considered excellent, according to the Brazilian Guidelines on Arterial Hypertension^13^. However, in relation to AC, the highest mean was found in young people who knew their family history for stroke. While the blood pressure levels undergo change more slowly, the measurement of AC is more sensitive to change resulting from lifestyle and the ingestion of foods which are higher in calories. Although this difference is present, both the classification of arterial blood pressure and of AC presented statistical association with knowledge regarding the history of stroke in the family. 

Nevertheless, although this age range presents, in the majority of the sample, adequate blood pressure levels, many young people presented systolic arterial hypertension (SAH), which increases cardiovascular risk. This fact should not be overlooked or treated superficially by the health services^18^. In its turn, obesity increases the risk of developing stroke ^19^, regardless of age, as it is related to higher incidence of obstructive sleep apnea in this population. It is associated with fragmentation of sleep, sleepiness and hypoxemia, increasing the risk of stroke, even in young people^20^. In this case, young people who are obese should concern themselves even more with finding out their family history, so that they can adopt preventive measures. 

Interventions undertaken in youth, which is a critical period for developing various risk factors, are recommended as a means of avoiding outcomes in adult life^21^. One can perceive the need for studies directed towards the young adult population, and that educational programs should be increased, coming to form part of educational measures geared towards this population, such that this topic should be known and that raising the issue of the family history should come to be something more routine in the family's dialogue.

As a result, further studies are recommended on the issue, with a random sample, given that one limitation of the present study was the sample by convenience, although the number of subjects was appropriate. Furthermore, this study's limitations are also related to its design and to its power of generalization, as the transversal approach makes it difficult to establish the causal relationship between the variables. 

## Conclusion

The young adults' knowledge regarding family history of stroke was shown to be associated with the following factors: civil status, blood pressure levels and AC measurement. The sample by convenience, and the transversal design adopted, were shown to be limitations of the study. 

Nevertheless, the variables tested reinforce the need for intervention on the part of nurses and other health professionals in relation to the young adults, given that knowledge regarding family history of stroke can support health education, with a view to the adopting of a healthy lifestyle. This could take place in the healthcare networks in which the young are inserted, given that these are shown to represent a group which is vulnerable to adopting behaviors which increase the risk of stroke. 
